# PP02 Analysis Of The Logistics And Time Impact Of Different Treatments Available For Paroxysmal Nocturnal Hemoglobinuria In Brazil

**DOI:** 10.1017/S0266462325102225

**Published:** 2025-12-29

**Authors:** Verónica Mata, Laura Branco, Florine Cordeiro

## Abstract

**Introduction:**

Paroxysmal nocturnal hemoglobinuria (PNH) is a rare and life-threatening blood disorder, affecting one to 1.5 new individuals per million annually worldwide. In Brazil, there are three registered treatments: eculizumab, pegcetacoplan, and ravulizumab, while crovalimab is currently under evaluation by ANVISA, the Brazilian Health Regulatory Agency. This study examined the impact of these PNH treatments on the logistics and time required by healthcare professionals and patients for administration.

**Methods:**

Quantitative and descriptive analysis was conducted based on information from the package leaflets of eculizumab, ravulizumab, pegcetacoplan, and crovalimab regarding the number of vials, volume, and administration time for each medication for a patient with PNH weighing between 60 and 100 kg over a period of 52 weeks.

**Results:**

The number of vials required for treatment over 52 weeks was 78 for eculizumab, 102 for pegcetacoplan, 70 for ravulizumab, and 26 for crovalimab. The volume to be transported would be 4,680 mL for eculizumab, 2,080 mL for pegcetacoplan, 231 mL for ravulizumab, and 52 mL for crovalimab. Regarding the time required for each infusion per year (52 weeks), it would take 14.6 hours for eculizumab, 4.7 hours for ravulizumab, 78 hours for pegcetacoplan, and 1.1 hours for crovalimab.

**Conclusions:**

This study demonstrated that crovalimab can reduce the number of vials needed by 169 percent, transportation and storage volume by 344 percent, and infusion time by 331 percent. These efficiencies, applicable to both healthcare professionals and patients, highlight crovalimab’s potential to significantly enhance the sustainability of Brazilian public healthcare system and improve quality of life for patients.

